# Supplementary material for: Knowledge, Attitude and Practice Regarding Antibacterial and Their Resistance in Medellín-Colombia: A Cross-Sectional Study
Source: Antibiotics (Basel). 2023 Jun 25;12(7):1101. doi: 10.3390/antibiotics12071101 (PMC10376581; doi:10.3390/antibiotics12071101)
Supplement: Supplementary file 1 [file antibiotics-12-01101-s001.zip › antibiotics-2414732-supplementary.pdf]

**Supplementary material**

**Table S1.** Multiple comparisons were adjusted using the Bonferroni test

| | **Knowledge** | **Attitudes** | **Practices** |
|---|---|---|---|
| Older adult -Youth | 0.162 | 0.976 | 0.030 |
| Older adult- Adult | <0.001 | 0.020 | 0.007 |
| Youth-Adult | 0.135 | 0.565 | 1.000 |
| **Economic status** | | | |
| Low-Medium | <0.001 | 0.004 | 0.004 |
| Low-High | 0.012 | 0.426 | 0.718 |
| Medium-High | 0.835 | 1.000 | 1.000 |
| **Education level** | | | |
| None- Primary school | 1.000 | 0.932 | 1.000 |
| None- High school | 0.293 | 0.074 | 1.000 |
| None- Technical school | 0.001 | 0.004 | 1.000 |
| None- College student | <0.001 | <0.001 | 1.000 |
| None- Professional | <0.001 | <0.001 | 1.000 |
| Primary school- High school | 0.026 | 0.232 | 0.315 |
| Primary school- Technical school | <0.001 | <0.001 | 1.000 |
| Primary school- College student | <0.001 | <0.001 | 1.000 |
| Primary school- Professional | <0.001 | <0.001 | 1.000 |
| High school- Technical school | <0.001 | 0.379 | 1.000 |
| High school- College student | <0.001 | 0.012 | 0.084 |
| High school- Professional | <0.001 | 0.001 | 0.026 |
| Technical school- College student | 1.000 | 1.000 | 1.000 |
| Technical school- Professional | 0.069 | 1.000 | 1.000 |
| College student- Professional | 1.000 | 1.000 | 1.000 |

**Table S2.** Description of the Psychometric Properties of the Instrument

| **Knowledge** | **Item-Index correlation** | **Attitudes** | **Practices** |
|---|---|---|---|
| Antibiotic resistance is very dangerous to human health | 0.541** | 0.208** | 0.027** |
| Penicillin is an antibiotic | 0.376** | 0.135** | 0.044** |
| If antibiotics are consumed too constantly or misused, they may stop working | 0.504** | 0.233** | 0.039** |
| er-the-counter use of antibiotics can cause bacteria to become resistant | 0.566** | 0.182** | 0.047** |
| When I take antibiotics without a prescription, I am contributing to bacterial resistante | 0.565** | 0.178** | 0.005** |
| Currently it can occur infections by bacteria with resistance to all antibiotics | 0.435** | 0.102** | 0.064** |
| Antibiotic treatment can be stopped when you feel better | 0.504** | 0.481** | 0.347** |
| Antibiotics are the same drugs used to relieve pain and fever | 0.500** | 0.330** | 0.176** |
| Bacterial resistance is a serious problem in other countries, not here in Colombia | 0.403** | 0.300** | 0.226** |
| Resistant infections only affect people in hospitals or with severe illnesses | 0.449** | 0.372** | 0.202** |
| Floor effect | 0.1% | | |
| Ceiling effect | 5.2% | | |
| Internal consistency success % | 90% (9/10) | | |
| Discriminating power success % | 100% (20/20) | | |
| Cronbach's Alpha | 0.701 | | |
| **Attitudes** | **Item-Index correlation** | **Knowledge** | **Practices** |
| The community should receive more information about antibiotics and bacterial resistance | 0.416** | 0.263** | 0.102** |
| Everyone should be aware of the importance of using antibiotics correctly | 0.449** | 0.279** | 0.103** |
| I am concerned about the impact that bacterial resistance may have on my health and the heath of my family | 0.414** | 0.254** | 0.036** |
| I Should advise family and friends not to take antibiotics without a prescription | 0.523** | 0.254** | 0.181** |
| People should use antibiotics only when prescribed by a physician | 0.553** | 0.253** | 0.236** |
| I expect my doctor to prescribe antibiotics if I have common cold symptoms (cough/flu) | 0.470** | 0.175** | 0.166** |
| Antibiotic treatment should be stopped as soon as symptoms or discomfort disappear | 0.625** | 0.455** | 0.361** |
| A doctor who does not prescribe antibiotics when the patient thinks he should is a bad doctor | 0.510** | 0.270** | 0.236** |
| It is okay to save antibiotics from a previous treatment for later use | 0.475** | 0.163** | 0.320** |
| It is good to be able to buy antibiotics without a prescription | 0.594** | 0.229** | 0.347** |
| Floor effect | 0.1% | | |

| | | | |
|---|---|---|---|
| Ceiling effect | 12% | | |
| Internal consistency success % | 100% (10/10) | | |
| Disciminating power success % | 100% (20/20) | | |
| Cronbach's Alpha | 0.706 | | |
| **Practices** | **Item-Index correlation** | **Attitudes** | **Knowledge** |
| Have you ever taken antibiotics left over from a previous illness? | 0.454 | 0.202 | -0.010 |
| Did the pharmacy recommend you to take antibiotics? | 0.628 | 0.219 | 0.029 |
| Antibiotics are easy to obtain in pharmacies, you get them when you need them without a prescription | 0.606 | 0.161 | 0.035 |
| Have you ever changed of modified the doses of antibiotic treatment? | 0.425 | 0.176 | 0.021 |
| Have you ever taken an antibiotic for the common cold? | 0.630 | 0.327 | 0.230 |
| Do you advise others to take antibiotics? | 0.535 | 0.212 | 0.175 |
| Have you asked your doctor to prescribe antibiotics? | 0.453 | 0.239 | 0.175 |
| Have you ever taken antibiotics to prevent an illness from occurring? | 0.415 | 0.221 | 0.155 |
| Have you taken any steps to prevent antibiotics from stopping working in the future? | 0.466 | 0.208 | 0.210 |
| Have you taken any steps to protect family and friends from bacterial resistance? | 0.436 | 0.190 | 0.191 |
| Have you stopped taking antibiotics as soon as you start to feel better or symptoms disappear | 0.633 | 0.411 | 0.330 |
| Did you ever have the experience that the antibiotic was not effective? | 0.390 | 0.150 | 0.021 |
| Floor effect | 0.1% | | |
| Ceiling effect | 1.7% | | |
| Internal consistency success % | 92% (11/12) | | |
| Disciminating power success % | 100% (24/24) | | |
| Cronbach's Alpha | 0.706 | | |
